# The Effect of Maternal Blood Glucose on Umbilical Cord Blood Fibrinogen in Women With Gestational Diabetes

**DOI:** 10.7759/cureus.65020

**Published:** 2024-07-21

**Authors:** Sahar Jassim Abid, Thikra N Abdulla, Farah Sadiq

**Affiliations:** 1 Department of Obstetrics and Gynecology, Al-Kindy College of Medicine, University of Baghdad, Baghdad, IRQ; 2 Department of Obstetrics and Gynecology, Al-Elwiya Maternity Teaching Hospital, Baghdad, IRQ

**Keywords:** gynecology, fibrinogen, umbilical cord, women, gestational diabetes

## Abstract

Background and aims: Gestational diabetes mellitus (GDM) is delineated by the presence of glucose intolerance at any level that manifests or is initially identified during pregnancy. Factor I fibrinogen is among the most essential blood coagulation proteins. The concentration of fibrinogen influences platelet aggregation and blood viscosity. This study aimed to determine the correlation between fetal cord blood fibrinogen and plasma fibrinogen in pregnant women with GDM and between fetal cord blood fibrinogen and maternal blood sugar.

Methods: A cross-sectional study was executed at Al-Elwiya Maternity Teaching Hospital in the obstetrics and gynecology department. The sample included 90 term pregnant women: 45 were confirmed to have GDM, and 45 healthy pregnant women served as control. Estimation of prelabor maternal fasting and random plasma glucose and plasma fibrinogen was performed. During delivery, blood was drawn from the umbilical cord to estimate neonatal plasma glucose and fibrinogen levels.

Results: The mean maternal plasma fibrinogen level exhibited a notable increase in women with GDM compared to the control (330.11 ± 56.92 mg/dl versus 254.89 ± 41.01 mg/dl). The infants of diabetic mothers had significantly lower mean cord plasma glucose levels (65.71 ± 14.63 mg/dl versus 77.80 ± 7.81 mg/dl) and higher mean cord plasma fibrinogen levels (269.42 ± 25.91 mg/dl versus 229.69 ± 21.29 mg/dl). Umbilical cord plasma fibrinogen was correlated positively with maternal plasma sugar and fibrinogen.

Conclusion: A positive correlation between maternal and fetal cord fibrinogen levels was determined in women with GDM. Monitoring plasma fibrinogen levels in neonates of mothers with GDM could be facilitated by longitudinal, large-scale validation studies enabled by artificial intelligence as a new, evolving technique that contributes to more valuable outcomes. This would shed additional light on the course and function of plasma fibrinogen for a more comprehensive analysis of the fetal clotting system.

## Introduction

Gestational diabetes mellitus (GDM) refers to any glucose intolerance level that becomes apparent or is identified during pregnancy [1]. Based on the International Diabetes Federation, diabetes impacts one out of every six pregnancies (16.8%). The proportion of this population afflicted with pregestational diabetes is 13.6%, whereas GDM affects the majority (86.4%). Pregnant diabetes incidence differs by ethnicity and race [2]. Sedentary lifestyles, rising maternal age, and emerging obesity all contribute to the global increase in the incidence of GDM. In addition, the adoption of stricter diagnostic criteria set forth by the International Association of Diabetes and Pregnancy Study Groups (IADPSG) has played a role in the increase in its prevalence [1].

In the Middle East and North Africa (MENA) region, the pooled weighted prevalence of GDM is 13.0%. From 2010 to 2019, the prevalence of GDM was found to be the highest in Qatar (20.7%) and the lowest in Jordan (4.7%). Between the years 2000 and 2009, as well as 2010 and 2019, GDM increased by 4%-8% in Saudi Arabia and Oman, whereas it decreased by 2%-4% in Bahrain, Qatar, and the United Arab Emirates [3]. Among Middle Eastern nations, Iraq has a medium diabetes mellitus prevalence [4]. GDM prevalence is 7% worldwide, according to local reports [5]. During pregnancy and childbirth, untreated GDM is linked to detrimental consequences for the mother and fetus. These include preeclampsia, necrosis, birth injuries, fetal overgrowth, neonatal low blood sugar, and elevated bilirubin levels. Additionally, GDM is correlated with significant long-lasting effects; women who develop GDM face an increased risk of having type II diabetes mellitus, metabolic abnormalities, and cardiovascular complications in the future [6]. The placenta may have a significant impact on GDM pathophysiology, although the exact processes that cause the disease remain unknown [7].

Impaired glucose tolerance in diabetic individuals increases thrombogenic factors like fibrinogen [8]. Fibrinogen is an acute-phase reactant that elevates in response to systemic inflammation in the bloodstream, tissue damage, and other specific events [9]. High fibrinogen levels in diabetes, inflammation, and other disorders are believed to contribute to the development of blood clots and damage to blood vessels [6]. Various controllable and uncontrollable factors, such as age, gender, smoking, body mass index, hypertension, alcohol consumption, glycemic control, lipid profile, and urine albumin excretion rate, influence the amount of fibrinogen (8). Pregnancy induces a hypercoagulable condition as a natural and adaptive strategy to maintain hemostatic equilibrium and avoid excessive blood loss during childbirth [10]. This natural mechanism may become a pathological process in a pregnancy complicated by GDM [11]. During midpregnancy, women with GDM exhibited a notable rise in blood fibrinogen levels, which is significant compared to late pregnancy [12]. Neonates born to women with GDM had considerably higher plasma fibrinogen levels when they had sepsis compared to those without sepsis [13]. The study’s purpose was to determine if there is a correlation between fetal cord blood fibrinogen and plasma fibrinogen in expectant women with GDM and between fetal cord blood fibrinogen and maternal blood sugar.

## Materials and methods

A cross-sectional investigation was carried out at the obstetrics and gynecology department of Al-Elwiya Maternity Teaching Hospital from June 1, 2020, until the end of May 2021. The research protocol received approval from the institution's Administration Board and the Scientific Affairs Unit and Medical Ethics Committee at Al-Kindy College of Medicine (reference: 123/Feb. 3, 2020). All participants provided informed consent. A total of 90 term pregnant women with singleton viable fetuses were enrolled who were either healthy (control group) or had been diagnosed with GDM (study group). Patients with coagulation disorders, alternative forms of diabetes mellitus, medical conditions, obstetrical issues (Rh^-^ mother with Rh^+^ father, intrauterine death (IUD), preeclampsia, antepartum hemorrhage, and disseminated intravascular coagulation), neonatal complications or diseases, and preterm birth were excluded from the study.

The sample size was calculated by the following formula: n = 2(Z_α/2​_+Z_β_​)^2^σ2​/Δ^2^ where n = sample size per group; Z_α/2_​ = Z-value corresponding to the desired level of confidence (e.g., 1.96 for 95% confidence); Z_β_ = Z-value corresponding to the desired power (e.g., 0.84 for 80% power); σ = estimated standard deviation of the outcome variable; and Δ = minimum expected difference in the outcome variable between the two groups.

Group A comprised 45 pregnant individuals who had received a diagnosis of GDM antenatally with a documented oral glucose tolerance test (OGTT) that met the World Health Organization 2013 diagnostic criteria for GDM [14]. Group B consisted of 45 healthy pregnant women who served as control cases and had normal OGTT. All participants who attended the hospital were either in labor for vaginal delivery or were admitted for cesarean section (elective or emergency). A detailed history was taken, and a thorough general and obstetrical examination was performed by a resident doctor. Ultrasound and other investigations were reviewed to exclude any medical or obstetrical complications.

In the delivery room or surgical theater, 5 ml of venous blood was obtained from the pregnant women, and 5 ml of cord blood was collected from the newborns. The blood was stored in a plastic container with trisodium citrate as an anticoagulant. It was then separated into two 2.5 ml samples. One sample was centrifuged at 3000 rpm for 15 minutes to extract platelet-poor plasma, and the other was used for plasma glucose testing.

The Stago (STA)-fibrinogen assay kit provided by Diagnostica Stago, France, was utilized in conjunction with Stago STA R Coagulation Analyzer, provided by Diagnostica Stago analyzers to quantitatively measure fibrinogen levels in plasma using the Clauss clotting technique. The plasma fibrinogen was analyzed using a human fibrinogen total antigen assay enzyme-linked immunosorbent assay (ELISA) kit in the first tube with an automated coagulation analyzer. The normal maternal plasma fibrinogen range in the third trimester was 373-619 mg/dL [15], and in the newborn, it was 125-300 mg/dL [16]. Abnormal results are those outside of these ranges. Glucose was measured by the glucose oxidase method (GOM).

For the statistical analysis, data were analyzed utilizing the IBM SPSS Statistics for Windows, Version 26 (Released 2019; IBM Corp., Armonk, New York, United States). Descriptive statistics were shown as mean ± standard deviation for progressive variables and as percentages for frequencies. The normal distribution of the dataset was validated through Kolmogorov-Smirnov analysis. Multiple contingency tables were constructed, and appropriate statistical tests were used. The chi-square test was used for categorical variables, whereas the t-test was utilized for comparing the two groups’ means. Pearson correlation analysis was performed to ascertain correlations. Statistical significance was determined at a significance level (p-value) of less than 0.05.

## Results

Table 1 compares the mean values of various variables between Group A and Group B. The results indicate no statistically significant differences between the groups for any of the measured variables.

**Table 1:** Distribution of demographical data SD: Standard deviation p-value was considered significant <0.05

Variable	Group A				Group B				p-value
	Mean	SD	Min	Max	Mean	SD	Min	Max	
Age	29.56	5.77	19	41	31.27	6.68	20	42	0.239
Gestational age	37.29	1.17	35.71	39.57	37.68	1.09	35.85	39.28	0.07
Gravidity	3.20	1.56	1	7	3.20	1.65	1	8	0.984
Parity	1.67	1.37	0	6	1.56	1.29	0	6	0.757
Miscarriage (abortion)	0.53	1.01	0	4	0.64	0.96	0	3	0.325

The mean maternal fasting blood sugar (FBS) and 1 h random blood sugar (RBS) showed a high increase in Group A compared with the healthy control group (Group B); 14 (31%) of the diabetic patients had good glycemic control, and 31 (69%) had poor control. The mean maternal fibrinogen level was significantly higher in Group A (330.11 ± 56.92 mg/dl) than in Group B (254.89 ± 41.01 mg/dl). Details are shown in Table 2.

**Table 2:** Comparison of maternal plasma glucose and fibrinogen levels in the study groups FBS (mg/dl): Fasting blood sugar (milligrams per deciliter); 1 h RBS (mg/dl): one-hour random blood sugar (milligrams per deciliter); SD: standard deviation p-value was considered significant <0.05

Variable	Group A				Group B				p-value
	Mean	SD	Min	Max	Mean	SD	Min	Max	
FBS (mg/dl)	152.31	8.52	91	285.00	80.71	5.44	78	91	<0.001
1 h RBS (mg/dl)	200.73	6.07	104	334.00	90.98	3.50	83	98	<0.001
Plasma fibrinogen (mg/dl)	330.11	56.92	230	415	254.89	41.01	181	319	<0.001

The maternal plasma fibrinogen level of groups A and B was moderately correlated with FBS (r = 0.398) and 1 h RBS (r = 0.514). That was statistically significant, as shown in Table 3.

**Table 3:** Correlation among maternal plasma fibrinogen, FBS, and 1 h RBS FBS: Fasting blood sugar; 1 h RBS: one-hour random blood sugar p-value was considered significant <0.05

Variable		Plasma fibrinogen
FBS	Pearson correlation	0.398
	p-value	<0.001
1 h RBS	Pearson correlation	0.514
	p-value	<0.001

Regarding the neonates, the mean gestational age of Group A was 268.84 days ± 9.44, which was not significantly different than Group B, as shown in Table 3. The mean cord RBS was statistically significantly lower in Group A (65.71 ± 14.63 mg/dl) than that in Group B (77.80 ± 7.81 mg/dl). Additionally, the mean cord fibrinogen was significantly higher in Group A (269.42 ± 25.91 mg/dl) compared to that in Group B (229.69 ± 21.29 mg/dl); details are shown in Table 4.

**Table 4:** Descriptive analysis of the neonatal outcomes GA: Gestational age; C-RBS: cord random blood sugar; C-fib: cord fibrinogen level; mg/dl: milligrams per deciliter; SD: standard deviation p-value was considered significant <0.05

Variables	Group A				Group B				p-value
	Mean	SD	Min	Max	Mean	SD	Min	Max	
GA (days)	268.84	9.44	253	290	265.53	8.94	246	283	0.253
C-RBS (mg/dl)	65.71	14.63	44	89	77.80	7.81	66	92	<0.001
C-fib (mg/dl)	269.42	25.91	225	309	229.69	21.29	195	268	<0.001

A positive correlation was observed between umbilical cord fibrinogen and maternal FBS, 1 h RBS, and fibrinogen. In contrast, a weak inverse correlation was observed between umbilical cord RBS and maternal FBS and fibrinogen, as depicted in Table 5.

**Table 5:** Cord and maternal glucose and fibrinogen level correlation FBS: Fasting blood sugar; RBS: random blood sugar p-value was considered significant <0.05

Variables		Maternal		
		FBS	RBS	Fibrinogen
Fetal cord fibrinogen	Correlation	0.447	0.362	0.246
	p-value	<0.001	<0.001	0.019
Fetal cord RBS	Correlation	-0.287	-0.184	-0.225
	p-value	0.006	0.083	0.033

## Discussion

A rise in the prevalence of GDM may indicate a rise in diabetes and obesity rates. Thus, the public health implications of rising GDM rates need more focus, in addition to the negative effects on newborns [17]. This study examined the association between maternal and fetal hyperglycemia and fibrinogen levels. Both placental and neonatal blood samples can be used to conduct routine coagulation tests in newborns [18]. Therefore, changes in the level of cord fibrinogen, type I diabetes mellitus, type II diabetes mellitus, hypertension, cigarette smoking, and hypercholesterolemia may reflect changes in the neonatal coagulation cascade, highlighting the possibility of increased thrombotic complications.

We observed a significantly higher fibrinogen level in maternal and fetal blood when the mother had GDM compared to the healthy control group. Several investigations have shown a considerable increase in blood fibrinogen levels in women with GDM, as reported by Mahjabeen et al. [19], Abdel Gader et al. [20], and Di Benedetto et al. [21]. Similar findings were also reported in individuals with type I and II diabetes mellitus [22,23]. We observed that the fibrinogen increase was moderately associated with 1 h RBS, while others reported a moderate association with the fasting insulin level [19]. It is established that GDM may predispose patients to blood clotting due to heightened platelet activation, increased production of coagulation factors such as fibrinogen, and decreased fibrinolytic activity [10]. Research has shown that individuals with GDM had a greater fibrinogen level compared to those without GDM, and hyperfibrinogenemia increased each trimester [10]. Nevertheless, a distinct alteration within proinflammatory levels of cytokine (IL-2, IL-6, and IL-8) was not identified, suggesting a potential alternative explanation for pregnant hyperfibrinogenemia aggravated by GDM other than inflammation [20].

In the current research, the cord RBS level exhibited a significant decrease in neonates delivered to mothers with GDM compared to those born to healthy mothers. Studies by Arimitsu et al. [24], Alemu et al. [25], Voormolen et al. [26], and Zhao et al. [27] reported similar findings on newborn blood glucose levels, showing a higher incidence of neonatal hypoglycemia in individuals with GDM. We found a weak yet significant negative correlation between cord blood glucose and maternal FBS and serum fibrinogen. We found no significant link with maternal 1 h RBS.

The level of umbilical cord fibrinogen in the current study was found to be significantly higher in newborns whose mothers had GDM compared to those whose mothers were healthy. These findings have not been well studied in the existing literature; only Bashir et al. [28] addressed this topic and reported a comparable result. Further analysis showed that umbilical cord fibrinogen had a significant positive correlation with maternal serum fibrinogen, FBS, and 1 h RBS. Fibrinogen is an essential component of the common route of coagulation, and it plays a role in the acute-phase response following tissue injury and inflammation [9,29]. Previous research has reported an elevated level of fibrinogen in neonatal sepsis [13]. In the context of GDM, studies addressing neonatal fibrinogen are scarce. Hence, more studies are required to determine the long-term effects of fibrinogen on neonates in cases of GDM [30].

Several limitations exist within the research, the sample size was relatively small, with only 90 participants, which may not adequately represent the broader population of pregnant women with GDM. This limited sample size also affects the statistical power of the study and the ability to generalize the findings. Additionally, the study was conducted at a single center, which may limit the applicability of the results to other settings or populations. Further research with larger, more diverse populations and a longitudinal design is needed to confirm these findings and elucidate the underlying mechanisms.

## Conclusions

In conclusion, a positive correlation between maternal and fetal cord fibrinogen levels was determined in women with GDM. Monitoring plasma fibrinogen levels in neonates of mothers with GDM could be facilitated by longitudinal, large-scale validation studies enabled by artificial intelligence as a new, evolving technique that contributes to more valuable outcomes. This would shed additional light on the course and function of plasma fibrinogen for a more comprehensive analysis of the fetal clotting system.
